# Inhibition of EETosis with an anti-citrullinated histone antibody: a novel therapeutic approach for eosinophilic inflammatory disorders

**DOI:** 10.3389/fimmu.2025.1533407

**Published:** 2025-02-20

**Authors:** Eline Zwiers, Daphne Montizaan, Annemarie Kip, Kelsy Waaijenberg, Paul S. Fichtinger, Sameer K. Mathur, Yuki Fujioka, Shigeharu Ueki, Helmuth van Es, Renato G. S. Chirivi, Eric Meldrum, Maarten van der Linden

**Affiliations:** ^1^ Research & Development, Citryll B.V., Oss, Netherlands; ^2^ Division of Allergy, Pulmonary, and Critical Care, Department of Medicine, University of Wisconsin School of Medicine and Public Health, Madison, WI, United States; ^3^ Department of General Internal Medicine and Clinical Laboratory Medicine, Akita University Graduate School of Medicine, Akita, Japan

**Keywords:** eosinophil extracellular traps, EETosis, PAD4, citrullination, galectin-10, anti-citrullinated histone antibody, eosinophilic chronic rhinosinusitis

## Abstract

Eosinophils are a subset of granulocytes that protect the host against fungal and parasitic infection through secretion of their granular contents. In response to specific stimuli, eosinophils also undergo a type of lytic cell death, referred to as eosinophil extracellular trap (EET)-associated cell death (EETosis), where histone citrullination facilitates chromatin decondensation, cell rupture and release of pro-inflammatory, decondensed chromatin into the extracellular environment as EETs. In this study, we show the abundant presence of eosinophils and citrullinated histones in nasal polyp tissue of patients with eosinophilic chronic rhinosinusitis (ECRS). Using live imaging microscopy on purified human eosinophils, we demonstrate that physiologically relevant stimuli induce release of citrullinated EETs and the marker of eosinophil activation galectin-10. While the kinetics of release of EETs and galectin-10 are similar, inhibitors of citrullination block EETosis in a dose dependent manner but fail to inhibit galectin-10 release. The importance of citrullination is further exemplified with CIT-013, a monoclonal antibody specific for citrullinated histones H2A and H4. CIT-013 potently inhibits release of EETs (half-maximal inhibitory concentration of 2.5 nM) without inhibiting other eosinophil functions such as degranulation, adhesion, superoxide production and induction of chemokine expression. Together, this study provides new insights into the requirement of protein arginine deiminase 4 (PAD4) for EETosis, differentiates requirements of EETosis from galectin-10 release, and identifies a novel therapeutic approach for EETosis inhibition by targeting citrullinated histones in eosinophil-driven diseases such as ECRS.

## 1 Introduction

Eosinophils are innate immune cells belonging to the granulocyte subtype, characterized by intracellular granules which stain positive with eosin. In a healthy setting, eosinophils comprise 1-3% of circulating white blood cells and are present in tissue primarily at the mucosal sites of the gastrointestinal tract, lungs, and mammary glands (1). Eosinophils rapidly mediate immune responses against pathogens such as bacteria, fungi, viruses, and helminths by releasing the content of their granules via a selective release called piecemeal degranulation or by release of total granular content via compound exocytosis (2).

Granule proteins participate in the killing of pathogens by generating free radicals (e.g. eosinophil peroxidase, EPX), disrupting the integrity of the lipid bilayer membrane (e.g. major basic protein, MBP) and by their ribonuclease activity (e.g. eosinophil-derived neurotoxin, EDN and eosinophil cationic protein, ECP) (3). In addition to these cationic proteins, eosinophil granules also contain cytokines, chemokines, and growth factors. These cytokines (e.g. interleukin (IL)-4, IL-13, IL-6 and IL-25) can establish a T-helper cell type 2 immune response (4) while others, such as transforming growth factor beta (TGF-β) and matrix metalloproteinases (MMP), promote tissue remodeling (5).

In addition to degranulation, activated eosinophils can also rupture to release their chromatin into the extracellular environment in structures termed eosinophil extracellular traps (EETs). This occurs by an ordered program of events resulting in a cytolytic cell death termed EET-associated cell death (EETosis), in reference to the analogous process called neutrophil extracellular trap (NET)-associated cell death (NETosis), previously described in neutrophils (6, 7). EETs are characterized by the colocalization of extracellular nuclear deoxyribonucleic acid (DNA) with granule contents and intact granules (6, 8, 9). Numerous stimuli have been reported to trigger EETosis *in vitro*: phorbol 12-myristate 13-acetate (PMA) (6, 10, 11), calcium ionophore (A23187) (6, 10–12), monosodium urate (MSU) crystals (13), platelet activating factor (PAF) in combination with IL-5 or granulocyte-macrophage colony-stimulating factor (GM-CSF) (6), IL-5 in combination with C-C motif chemokine ligand 11 (CCL-11) (10), activated platelets (11), *Aspergillus fumigatus* (14, 15), and immunoglobulins (6, 10, 16). Activation of the EETosis cascade involves an ordered series of dynamic morphological changes within the eosinophil that require the activity of nicotinamide adenine dinucleotide phosphate (NADPH) oxidase (6) and protein arginine deiminase 4 (PAD4) (11). Activated PAD4 converts arginine residues into citrulline, mainly on the N-terminal tail of histones, leading to the loss of nuclear lobulation, chromatin decondensation and nuclear membrane disintegration. Subsequently, chromatin expands within the cytoplasm to result in plasma membrane rupture and release of EETs into the extracellular environment (8, 17).

EETs have pro-inflammatory characteristics and have been demonstrated in circulation and tissue of multiple non-infectious, allergic, pro-thrombotic, and inflammatory eosinophilic disorders. A disease often associated with the presence of EETs is eosinophilic chronic rhinosinusitis (ECRS), a subgroup of CRS characterized by patients developing nasal polyps. ECRS is associated with significant eosinophilic infiltration and local inflammation of the nasal cavity and sinuses (18–20). Immunohistochemical (IHC) staining of nasal tissue and allergic mucin from ECRS patients revealed abundant EETs (8, 9, 18, 21–25) which positively correlate with increasing clinical symptoms (22). Furthermore, EETosis in human ECRS tissue has been closely associated with the formation of Charcot-Leyden crystals (CLCs) (22, 26), hexagonal bipyramidal structures composed of the eosinophil protein galectin-10 that in turn also elicits inflammation (27–29). These data indicate that EETs may act as pathogenic factors for eosinophil-driven diseases like ECRS although there are currently no direct EETosis targeting therapies. An anti-inflammatory humanized monoclonal antibody, termed CIT-013, that targets the N-terminus of citrullinated histone H2A and H4, is in clinical development for immune-mediated inflammatory diseases. CIT-013 has been shown to both potently inhibit NET release and enhance phagocytic clearance of NETs by macrophages (30, 31). Targeting NETs by CIT-013 in this manner, significantly reduces inflammation in a range of preclinical models of neutrophilic inflammation (30–32). In Phase 1 clinical studies, CIT-013 has been demonstrated to be safe and well tolerated in healthy volunteers (33).

In this study, we show the significant presence of citrullinated histones H2A and H4 in tissue from ECRS patients. In eosinophils prepared from healthy donors, different physiological stimuli are shown to activate release of EETs which possess citrullinated histones regardless of the stimulus. Such EETosis responses are PAD4 dependent and potently inhibited by CIT-013 with no effect on other eosinophil functions, such as superoxide production, degranulation, and chemokine expression. Galectin-10 release overlaps with the kinetics of EETosis but is independent of citrullination. Our study provides new insights on the importance of PAD4-mediated citrullination during EETosis, differentiates requirements of EETosis from galectin-10 release, and identifies a novel therapeutic approach for EETosis inhibition by targeting citrullinated histones in eosinophil-driven diseases such as ECRS.

## 2 Materials and methods

### 2.1 Human material and written informed consent

All human materials were obtained from de-identified participants who provided their written informed consent to participate in this study. Venous blood from healthy donors was obtained from the Sanquin blood bank in Nijmegen, the Netherlands. Venous blood from allergic rhinitis patients was obtained from the University of Wisconsin, Madison, WI, USA according to protocols approved by their Health Sciences Institutional Review Board. Tissue samples from ECRS patients were obtained from Akita University Graduate School of Medicine.

### 2.2 Immunofluorescence staining on tissue of eosinophilic chronic rhinosinusitis patients

Tissue specimens were surgically obtained from patients with ECRS (34). All subjects were newly diagnosed and have not been used systemic steroids. For MBP staining, the deparaffinized sample slides were incubated with 0.1% proteinase K for 6 min. The slides were blocked by Perm/Wash buffer (BD Biosciences, 554723) containing 10% bovine serum albumin (BSA) for 30 min and then incubated with rabbit anti-MBP antibody (kind gift from Prof. Kita H, Mayo clinic) for 30 min at 37°C. AlexaFluor-594-conjugated goat anti-rabbit IgG (1:200; A11072, Life Technologies) were used for 30 min incubation at room temperature (RT).

For citrullinated histone staining, antigen retrieval was performed by incubation of the deparaffinized sample slides in Tris-EDTA buffer in a microwave oven for 15 min. The slides were then incubated with 10 µg/mL rabbit anti-citrullinated histone H3 monoclonal antibody (Abcam, Cambridge, ab5103) for 90 min at RT. AlexaFluor-488-conjugated goat anti-rabbit antibody (Life Technologies, A11008) was then added for 30 min at RT. Citrullinated histone H2A and H4 staining was performed by incubation with 50 µg/mL FITC-conjugated CIT-013 for 60 min at 37°C.

Isotype-matched control antibodies and Hoechst 33342 (1:5000, Invitrogen, H3570) were used in each experiment. Samples were mounted using Prolong Diamond (Life Technologies) and images were obtained using a LSM 980 confocal microscope and analyzed using ZEN software (Carl Zeiss). The section was counterstained by hematoxylin-eosin (H&E).

### 2.3 Eosinophil and neutrophil isolation from healthy individuals

Blood was collected in Vacutainer^®^ K2-EDTA tubes (BD, 367525) and within 2 hours, eosinophils were isolated using the negative selection EasySep™ Direct Human Eosinophil Isolation Kit (Stemcell, 19656), according to manufacturer’s instruction. For the experiments in which eosinophils were stimulated with immobilized immune complexes (imICs), blood was collected in Vacutainer^®^ lithium-heparin tubes (BD, 367526) and subsequently isolated using density gradient centrifugation and the Eosinophil Isolation Kit with LS Column (Miltenyi, 130-092-010). In short, blood was diluted 1:1 with 1x DPBS (Gibco, 14190-144) containing 0,5% (w/v) BSA (Roche, 10735108001) and 2 mM EDTA (VWR, E177). The granulocyte fraction was isolated with Ficoll-Paque Plus (Sigma Aldrich, 17-1440-03) density gradient centrifugation (600 x g for 30 min) followed by erythrocyte lysis with ammonium-chloride-potassium buffer containing 155 mM NH_4_Cl (Sigma Aldrich, A9434), 10 mM KHCO_3_ (Sigma Aldrich, 237205), and 0.1 mM Na_2_EDTA (Sigma Aldrich, E5134) (pH = 7.2) for 3 min at RT. Eosinophils were isolated from the granulocyte fraction according to the manufacturer’s instructions (with centrifugation at 350 x g). No differences were demonstrated on eosinophil phenotype and function upon the two isolation procedures (data not shown). After both isolation methods, eosinophils were washed and resuspended in RPMI1640 without phenol red (Thermo Fisher Scientific, 15230204) supplemented with 0.1% (v/v) BSA (Sigma Aldrich, A7979), 10 mM HEPES (Thermo Fisher Scientific, 15630080), and 50 U/mL penicillin and 50 µg/mL streptomycin (Thermo Fisher Scientific, 15070063) (further referred to as EET buffer).

Neutrophils were isolated using Ficoll density gradient centrifugation, as described previously (35). Isolated neutrophils were resuspended in RPMI 1640 without phenol red, supplemented with 2% FBS, 10 mM HEPES and 1 mM CaCl (VWR, E506) and 50 U/mL penicillin and 50 µg/mL streptomycin (further referred to as assay buffer).

Eosinophils were counted with a Bürker-Türk counting chamber and neutrophils were counted with the Cellometer Auto T4 Bright Field Cell Counter using 0.4% trypan blue solution (Thermo Fisher Scientific, 15250061). The purity of eosinophil and neutrophil fractions was confirmed by flow cytometry according to the protocols described below, with a purity cutoff of >85%.

### 2.4 Eosinophil isolation from allergic rhinitis patients

For the experiments focusing on eosinophil superoxide anion production, adhesion, degranulation, and monocyte chemotactic protein-1 (MCP-1) expression, eosinophils were isolated from allergic rhinitis patients. Subjects were between the age of 18 and 55 and diagnosed with allergic rhinitis with or without mild asthma. Peripheral blood was separated by density centrifugation on a Percoll gradient to isolate the granulocyte fraction as previously described (36). Eosinophils were isolated from the granulocyte fraction with negative selection using anti-CD16 (Miltenyi Biotec, 130-045-701), anti-CD14 (Miltenyi Biotec, 130-050-201), anti-CD3 (Miltenyi Biotec, 130-050-101), and anti-CD235a (Miltenyi Biotec, 130-050-501) magnetic beads and autoMACS PRO Seperator (Miltenyi Biotec). A purity cutoff of 99% was used.

### 2.5 Flow cytometry

To confirm purity of eosinophils isolated from healthy donors, isolated eosinophils were resuspended in 1x DPBS containing 1% (w/v) BSA and 0.1% (w/v) NaN_3_ (Sigma Aldrich, S2002) (referred to as FACS buffer) and incubated with 50x diluted Human TruStain FcX (Biolegend, 422302) for 15 min at RT. Subsequently, eosinophils were incubated with 0.05 µg/mL APC-conjugated mouse anti-human CD45 antibody (Clone HI30; Biolegend, 304037), 4 µg/mL APC-Cy7-conjugated mouse anti-human CD66b antibody (Clone G10F5; Biolegend, 305126), 3.2 µg/mL PE-conjugated mouse anti-human Siglec-8 antibody (Clone 7C9; Biolegend, 347104), and 1000x diluted Fixable Viability Dye 506 (eBioscience, 65-0866-14) for 30 min at RT. Eosinophils were centrifugated for 3 min at 400 x g and washed twice with FACS buffer. After the second wash, eosinophils were resuspended in FACS buffer and measured on the BD FACS Canto II (BD Biosciences) with BD FACSDiva software (version 8.0.1). Analysis of flow cytometry data was performed with FlowJo software (version 10.10.0). Gating strategy to determine eosinophil purity is described in **Supplementary Figure 1**. Neutrophil purity was determined as previously described (35).

To demonstrate CIT-013 binding, eosinophils were stimulated with 2 µM A23187 in the presence of 20 µg/mL HL488-CIT-013 or HiLyte™ Fluor 488-conjugated human anti-hen egg lysozyme antibody (isotype control antibody; HL488-cIgG) for 120 min. In the final 15 min of incubation, APC-conjugated mouse anti-human Siglec-8 antibody (Clone 7C9; Biolegend, 347106) was added at a final concentration of 8 µg/mL. Eosinophils were centrifugated for 3 min at 400 x g and resuspended in FACS buffer before acquisition.

### 2.6 Preparation of immobilized immune complexes

To generate imICs, a 96-well Nunc MaxiSorp plate (VWR, 735-0083) was coated with 10 µg/mL human serum albumin (HSA; Akron biotech, AK8214) in 1x DPBS at 4°C overnight. Subsequently, the wells were washed three times with 0.05% (v/v) Tween 20 (VWR, 663684B) in 1x DPBS (further referred to as wash buffer) and incubated with 10 µg/mL rabbit anti-HSA antibody (Sigma Aldrich, A4033) in wash buffer for 60 min at RT with gentle agitation (400 rpm). Finally, wells were washed three times with wash buffer and three times with 1x DPBS, before adding eosinophils.

### 2.7 Live imaging microscopy assay

Quantitative immunofluorescence live imaging microscopy was used to visualize EETosis using a protocol as described previously (35) with minor adaptations. In short, eosinophils were resuspended in EET buffer and seeded at 2 x 10^4^ cells per well on clear flat-bottom black 96-well plates (Corning, 3603) pre-coated with 0.001% poly-L-lysine (Thermo Fisher Scientific, 15230204). Eosinophils were stimulated to induce EETosis with either 2 µM A23187, 40.5 nM PMA, 0.25 µM PAF with or without thrombin at indicated concentrations, or imICs, in the absence or presence of 169.3 nM CIT-013 or human anti-hen egg lysozyme antibody (isotype control antibody (cIgG); CrownBio, C0001-5). When concentrations of EETosis stimuli or antibodies deviate from the above, it is stated in the figure legends. A full-length non-good manufacturing practice CIT-013 pilot batch (P3807623) was produced by Lonza using their proprietary GS XceedTM gene expression system in combination with GS-Chinese ham­ster ovary cells (30).

To study EETosis signaling pathways, eosinophils were pre-incubated for 30 min at 37°C with indicated concentration of PAD2 inhibitor AFM-30a hydrochloride (MedChemExpress, HY-125099A) or PAD4 inhibitors JBI-589 (MedChemExpress, HY-153450), GSK484 (MedChemExpress, HY-100514), or BMS-P5 (MedChemExpress, 33581) before addition of the stimulus.

Sytox™ Green (Thermo Fisher Scientific, S7020) was added to the wells in a final concentration of 20 nM to visualize the EETs. EET release was recorded over time with the IncuCyte^®^ S3 platform at 37°C and 5% CO_2_ using a 20x objective. Phase contrast and green fluorescence (Ex/Em: 504 nm/523 nm, 200 ms exposure) images were taken at 4 spots per well.

### 2.8 Quantification of live imaging microscopy

Analysis of EET release was performed using IncuCyte^®^ software (version 2021A or version 2022B) and EETs were defined as Sytox™ Green positive areas larger than cells. Sytox™ Green positive pixels were identified in the green channel using adaptive segmentation and a threshold GCU of 4-7. The edge split tool was turned on with an edge sensitivity between -20 and 0 for accurate quantification of closely spaced objects. “Hole fill” was set on 100 µm^2^ and “adjust size” was set between -1 and 1 pixel. Filters were applied to only include objects with a minimum area of 200-300 µm^2^, and a mean intensity of maximal 17-50 GCU. If needed, additional settings were used to reduce false positives, including minimum mean intensity between 15-20 GCU, minimum eccentricity between 0.30-0.35, maximum centricity of 0.90-0.97, or maximum area of 1000 µm^2^. Analysis definitions were optimized for each individual experiment with filter settings in the above-described range. Raw data of the number of EETs per image was used for further calculations.

Number of cells per image was determined in the phase contrast images taken at 0 or 1 hour after stimulation. Segmentation was set at 0 to separate neighboring cells, size of the area was adjusted with 0-1 pixel, “hole fill” was set at 0-100 µm^2^, and “minimum area” was set at 50-100 µm^2^. Large cell aggregates or artifacts were excluded by setting a maximum area of 600-3000 µm^2^ and/or a maximum eccentricity of 0.9-0.98.

The percentage of EETs was determined by dividing the number of EETs by the number of cells per image x 100%.

### 2.9 Detection of eosinophil-derived neurotoxin and galectin-10 release

Eosinophils were plated and stimulated with EETosis-inducing stimuli as described before, in the absence or presence of 169.3 nM CIT-013 or cIgG. No Sytox™ Green was added to the wells. Supernatants were collected at 15 min, 1 hour, and 3 hours after stimulation and were centrifuged at 300 x g for 5 min to remove cell debris.

EDN and galectin-10 in the supernatants were measured using the human EDN ELISA Kit (Hycult Biotech, HK391-02) and galectin-10 ELISA kit (Invitrogen, EH204RB), respectively, according to manufacturer’s protocol. Absorbance was measured at 450 nm (with 620 nm reference) using a Tecan Infinite F50 plate-reader controlled by Magellan F50 software (version 7.2).

### 2.10 EET harvest and citrullinated nucleosome ELISA

Eosinophils were plated and stimulated with EETosis-inducing agents as described above, for 4 hours at 37°C and 5% CO_2_ in the absence of Sytox™ Green. The wells were washed once with assay buffer before EETs were digested with 15 U/mL micrococcal nuclease solution (Thermo Fisher Scientific, EN0181) in assay buffer for 15 min at 37°C and 5% CO_2_. Subsequently, EDTA (pH = 8.0; VWR, E177) was added up to a final concentration of 9.8 mM to stop nuclease activity. Supernatants were transferred to a V-bottom 96-well plate (Greiner, 651101) and centrifuged at 20 x g for 5 min at RT to pellet cell debris.

The DNA concentration of harvested EETs was determined using the Quant-iT PicoGreen dsDNA Assay kit (Invitrogen, P11496), according to manufacturer’s instructions.

As a control, DNA was isolated from unstimulated eosinophils using the QIAmp DNA Blood Mini kit (Qiagen, 51104) according to manufacturer’s instruction. The DNA concentration was determined using NanoDrop ND-1000 (Thermo scientific). Citrullinated nucleosome content in 2 ng eosinophil DNA was determined by ELISA as described elsewhere (30).

### 2.11 Eosinophil superoxide anion production

Eosinophils were cultured at 0.5 x 10^6^ cells/mL in Hank’s-balanced salt solution (HBSS; Corning, 21-022-CM) containing 0.1% gelatin (Sigma Aldrich, G-9382) and 1.2 mg/mL ferro-cytochrome C (Sigma Aldrich, C7752) at 37°C and stimulated with 10 ng/mL IL-5 (R&D, 205-IL-005), as previously described (37). To assess the effect of CIT-013 on IL-5-induced release of reactive oxygen species, 169.3 nM CIT-013 or cIgG was added. As control, 10 µg/mL of the anti-IL-5 receptor antibody benralizumab (MedChemExpress, HY-P9923) was used. Superoxide anion production was measured every 10 min for up to 2 hours by colorimetric change at 550 nm. Afterwards, 20 µg/mL superoxide dismutase (Sigma Aldrich, S7571) was added to remove all the superoxide from the samples serving as blanks. The superoxide anion production was reported as nmol of cytochrome C reduced per 1 x 10^6^ cells per minute, calculated as previously described (37).

### 2.12 Eosinophil adhesion

Eosinophils were resuspended at 0.1 x 10^6^ cells/mL in HBSS with 0.3% gelatin and added to wells with or without coating of 5 µg/mL vascular cell adhesion molecule (VCAM)-1 (R&D Systems, 809-VR-050). Subsequently, eosinophils were cultured for 30 min at 37°C in the presence of 10 ng/mL IL-5. Benralizumab, CIT-013, or cIgG were added to assess the ability to block IL-5-induced adhesion. Wells were washed and the remaining adherent eosinophils were cultured in 55 mM Tris buffer (pH = 8.0; Sigma Aldrich, T-8442) with 1 mM H_2_O_2_ (Sigma Aldrich, H-1009), 1 mM o-phenylenediamine HCl (Sigma Aldrich, P-1526), and 0.1% Triton X-100 (Sigma Aldrich, X-100) for 30 min at RT, as described previously (38). EPX activity was measured as a colorimetric change at 490 nm. Eosinophil adherence was measured as a percentage of EPX activity after rigorous washes compared to the total activity from the number of cells initially added to the wells (38, 39).

### 2.13 Eosinophil degranulation

Eosinophils were cultured at 1 x 10^6^ cells/mL and stimulated with 10 ng/mL IL-5. Benralizumab, CIT-013, or cIgG were added to assess the ability to block IL-5-induced degranulation. Cultures were incubated for 4 hours at 37°C and 5% CO_2_ as previously described (40). Cell-free supernatants were collected and stored at −80°C. EDN protein levels were measured by ELISA per manufacturer protocol (MBL International, 7630).

### 2.14 Monocyte chemotactic protein-1 qPCR

After collection of supernatants to assess EDN degranulation, eosinophils were lysed, and total RNA was purified using RNeasy columns (Qiagen, 74106) and cDNA was made using GoScript reverse transcriptase (Promega, A5004). Equal volumes of cDNA from each sample were added to a solution of 1x SYBR green master mix (Applied Biosystems, A25742) and 0.4 µM primers (**Table 1**) (obtained from Integrated DNA Technologies) and levels of MCP-1 mRNA were quantified using qPCR (Applied Biosystems Step One plus). Gene expression was reported as fold induction of control housekeeping gene Cyclophilin A.

**Table 1:** Primers used in qPCR experiments.

MCP-1 Forward	5’-CCTTACTTCCACCGACGATAC-3’
MCP-1 Reverse	5’-TGGTGAAGTTATAACAGCAGGTGACT-3’
Cyclophilin A Forward	5’-GCCGAGGAAAACCGTGTACT-3’
Cyclophilin A Reverse	5’-TGTCTGCAAACAGCTCAAAGG-3’

### 2.15 Live imaging immunofluorescence confocal microscopy

Eosinophils were washed twice with 1x DPBS, followed by staining with the PKH26 Red Fluorescent Cell Linker Kit for General Cell Membrane Labeling, according to manufacturer’s instructions (Sigma Aldrich, MIDI26-1KT), at a concentration of 0.4 µM PKH26 per 2 x 10^6^ eosinophils/mL. Subsequently, eosinophils were washed twice with EET buffer and resuspended in EET buffer containing 1 µM SPY-650 DNA dye (Tebu-Bio, SC501). After incubation for 45 min at 37°C, eosinophils were seeded at 2 x 10^4^ cells per well in poly-L-lysine pre-coated clear flat-bottom black 96-well plates. Eosinophils were stimulated with A23187 in the absence or presence of 25 µg/mL HL488-cIgG or HL488-CIT-013. Binding of CIT-013 to stimulated eosinophils was recorded over time at 37°C and 5% CO_2_ with an Olympus inverted IX83 microscope equipped with NL5+ with a laser combiner 4ln (405, 488, 561, 683 nm, 20mW each) and Hamamatsu camera Orca flash 4 v3, operated by Confocal.nl.

Every 3 min, images were taken from the green (Ex/Em: 504 nm/523 nm), orange (Ex/Em: 550 nm/567 nm), and red (Ex/Em: 652 nm/674 nm) fluorescent channels. Images were processed with ImageJ/Fiji software (version 2.14.0/1.54f).

### 2.16 Statistics

GraphPad Prism 10 software (version 10.2.0) was used for both generation of graphs and statistical analysis. Data is presented as mean ± standard error of the mean (SEM), as dot plots with median, or as box and whisker plots with minimum and maximum value. The sample size, indicated in the figure legends, was determined based on the robustness of the signal and the variation of the datapoints. No outliers were excluded. Differences were considered significant at *P*<0.05. The normal distribution of each data set was assessed with the Shapiro-Wilk test. Statistical significance was determined with different tests according to the number of groups, Gaussian distribution, and (un)paired data, as indicated in the figure legends. The half-maximal inhibitory concentration (IC_50_) was determined using non-linear regression analysis.

## 3 Results

### 3.1 Abundant citrullinated EETs in tissue of eosinophilic chronic rhinosinusitis patients

**Figure 1:**
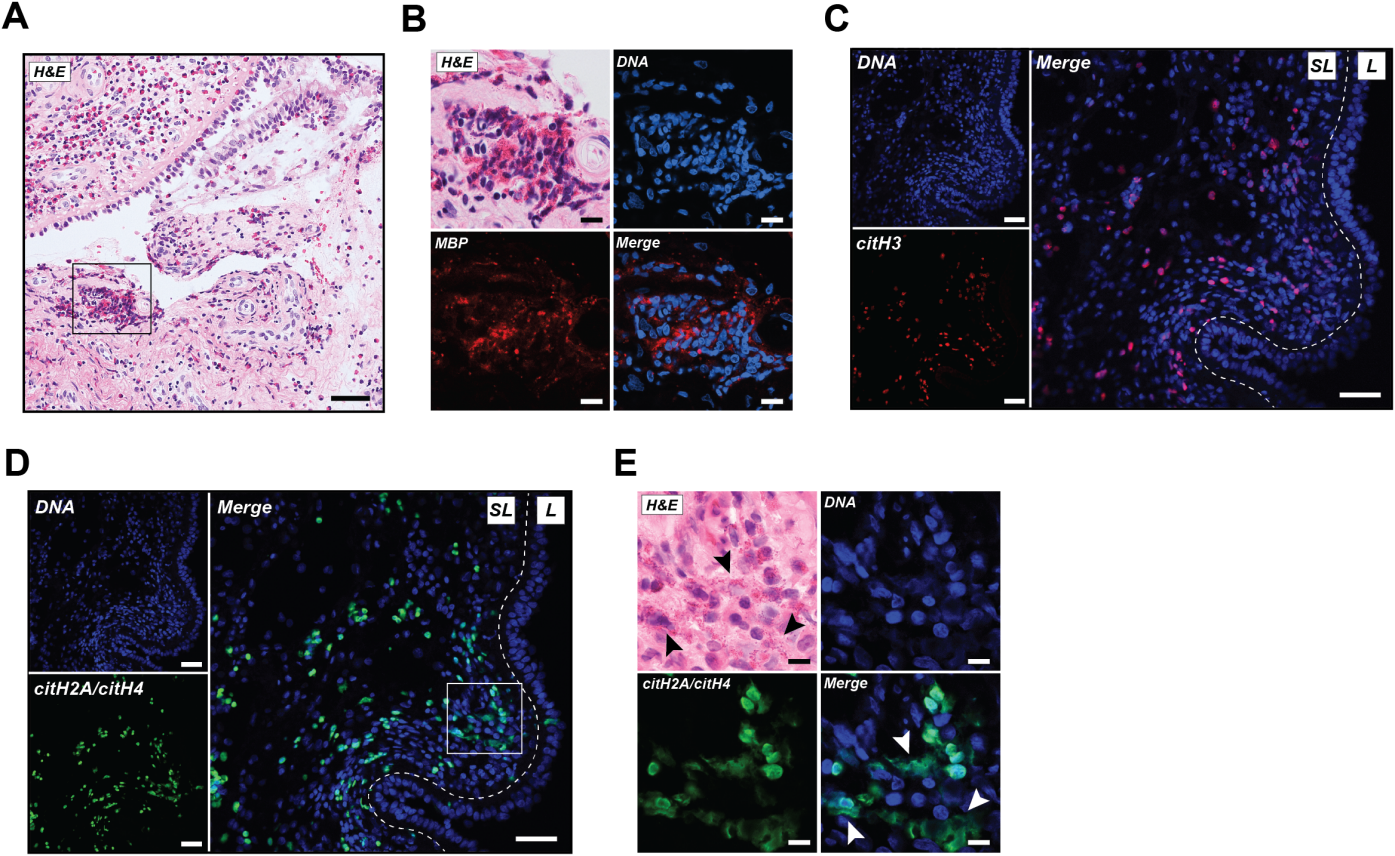
EETs rich in citrullination are abundantly present in human ECRS tissue. **(A)** A representative image of nasal polyp tissue stained with hematoxylin and eosin (H&E). **(B)** Increased magnification of selected area from A representing H&E stain, DNA (blue), and major basic protein (MBP; red). **(C)** Immunofluorescence images showing DNA (blue) and citrullinated histone H3 (citH3; red). **(D)** Immunofluorescence images from the same section as shown in **(C)** with DNA (blue) and CIT-013 epitope citrullinated histone H2A (citH2A) and citH4 (green). **(E)** Increased magnification of selected area from **(D)** representing H&E stain, DNA (blue), and citH2A and citH4 (green). White arrowheads indicate CIT-013 epitope colocalization with diffuse extracellular DNA, while black arrowheads indicate the colocalization of diffused extracellular DNA with the observed presence of eosinophils. Scale bars in A, C and D are 50 µm and in B and E are 10 µm. SL, Sublining; L, Lining.

To characterize the citrullination status of EETs in ECRS tissue, we performed IHC staining for the presence of citrullinated histones in nasal polyp biopsies of five ECRS patients. H&E staining showed a characteristic inflamed pattern with infiltration of leukocytes, predominately eosinophils, in the nasal polyp (**Figure 1A**), confirmed by the presence of the eosinophil-specific marker MBP (**Figure 1B**). Citrullinated histone H3 (citH3), a precedented marker for EETs in tissue (9, 11, 23, 24), was detected in the sublining of the tissue and confirms the presence of citrullinated EETs in ECRS (**Figure 1C**). Little staining was detected with the rabbit cIgG (**Supplementary Figure 2A**).

The potential of CIT-013 as therapy for ECRS, was assessed by performing IHC staining for the presence of CIT-013’s epitope, citrullinated histones H2A and H4, in ECRS tissue. CIT-013 epitope was detected mainly within the sublining of nasal polyp tissue (**Figure 1D**, **Supplementary Figure 2B**), while no staining was observed for the negative control human cIgG (**Supplementary Figure 2C**). Staining of CIT-013 epitope showed significant colocalization with diffused extracellular DNA (white arrowheads) and was positively correlated with the observed presence of cells morphologically consistent with being eosinophils (black arrowheads; **Figure 1E**). Together, these data highlight the abundance of citrullinated EETs in nasal polyp tissue of ECRS patients.

### 3.2 Kinetics and magnitude of EET release is dependent on the stimulus

**Figure 2:**
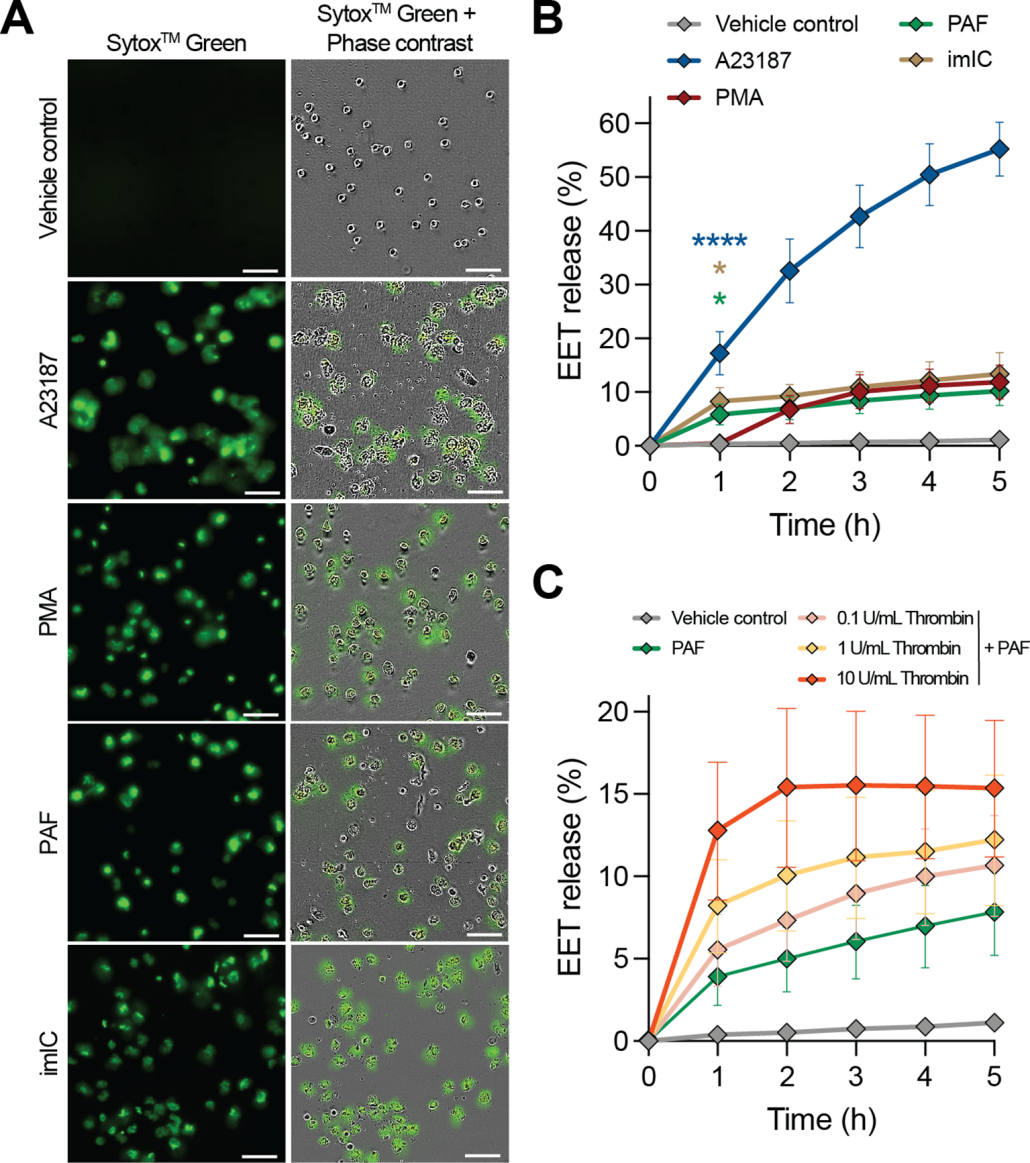
The kinetic profile of EET release using different EETosis-inducing stimuli visualized and measured by a quantitative immunofluorescence live imaging assay using the impermeable DNA dye Sytox^TM^ Green. **(A)** Representative images of EET release at 3 hours post stimulation with 2 µM A23187, 40.5 nM PMA, 0.25 µM platelet activating factor (PAF), and immobilized immune complexes (imICs). Scale bars are 50 µm. **(B)** Quantification of EET release over time upon stimulation with different stimuli (*n* = 4-12). **(C)** Quantification of EET release over time in the presence of 0.25 µM PAF in combination with the indicated concentrations of thrombin (*n* = 5-12). *P<0.05 and ****P<0.0001, Kruskal-Wallis test and Dunn’s multiple comparison test performed at t = 1 h.

Next, we investigated the EETosis response to various physiological stimuli in eosinophils purified from the blood of healthy volunteer donors. A previously described quantitative live imaging microscopy method was used to study the kinetics of EET release and the pharmacology of EETosis inhibitors (30, 35). Briefly, as previously demonstrated for the release of NETs by neutrophils, the plasma membrane impermeable DNA dye Sytox™ Green was used to visualize EET release from eosinophils. Eosinophils were stimulated to release EETs with A23187, PMA, PAF, and imICs (**Figure 2A**). The earliest timepoint at which EET release was detected was 1 hour after stimulation with A23187, imICs, or PAF while PMA results in detectable EET release 2 hours after stimulation (**Figure 2B**). The percentage of EET releasing cells was dependent on the stimulus concentration (**Supplementary Figure 3A**).

To understand whether EET release was altered in the presence of additional thrombosis-related factors, we tested PAF in combination with thrombin. While thrombin alone did not induce EET release (**Supplementary Figures 3B**), PAF-induced EET release was significantly elevated by thrombin in a concentration-dependent manner without changing the kinetic profile (**Figure 2C**; **Supplementary Figure 3C**). Enhanced EET release by the combination of thrombosis-related factors highlights the physiological relevance of EETosis in thrombotic diseases.

### 3.3 EETosis is dependent on PAD4 and EETs are rich in citrullinated histones

**Figure 3:**
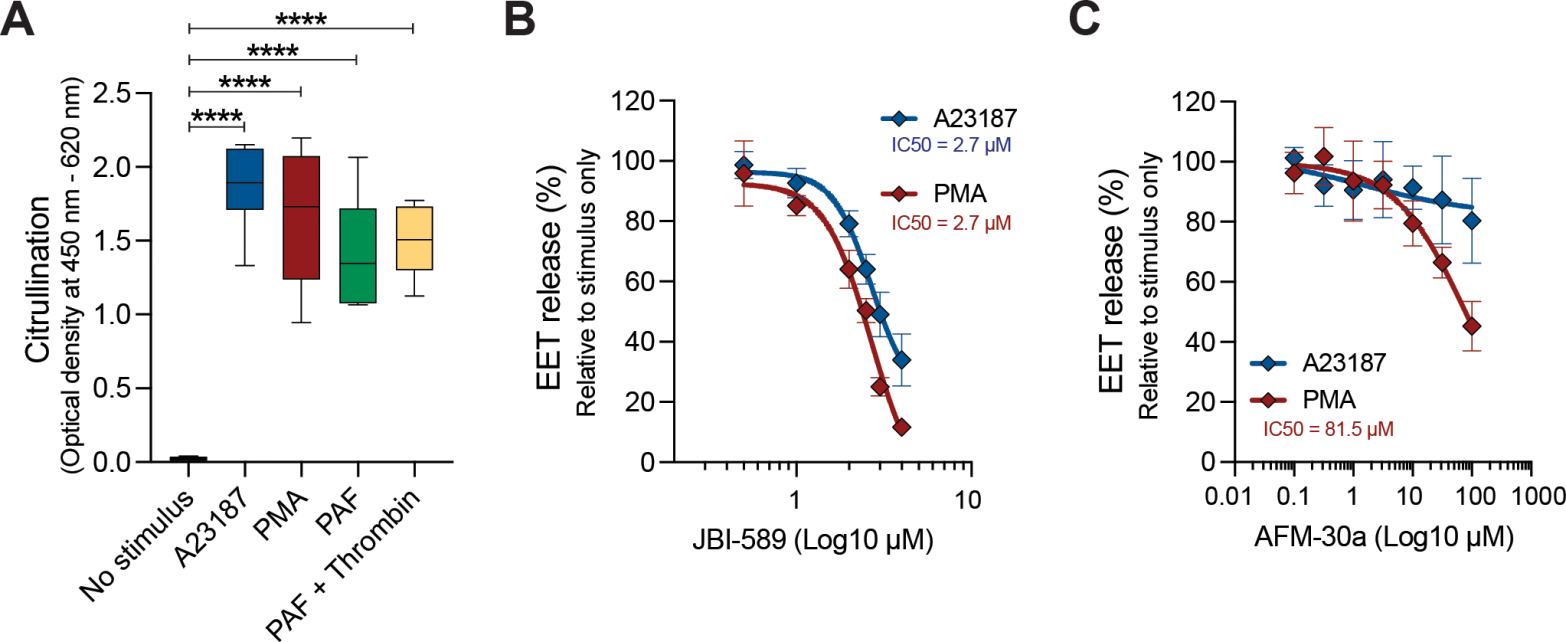
Histone citrullination is required for EETosis. **(A)** Citrullinated nucleosome detection in EETs harvested 3 hours post stimulation with 2 µM A23187, 40.5 nM PMA, and 0.5 µM platelet activating factor (PAF) without or with 1 U/mL thrombin (*n* = 2-4). Quantification of A23187- and PMA-induced EET release at t = 3 h in the presence of different concentrations of JBI-589 **(B)** and AFM-30a **(C)** (*n* = 4-7). Data were normalized to A23187- or PMA-induced EET release without inhibitor (set as 100% EET release). ****P<0.0001, ordinary one-way ANOVA with Dunnett’s multiple comparison test.

To investigate the importance of citrullination in EET release, we first investigated the presence of citrullinated nucleosomes within EETs using an ELISA that detects complexes of citrullinated histones H2A, H3 and H4 (30). Citrullinated nucleosomes were detected in EETs released upon stimulation with A23187, PMA, PAF, and a combination of PAF and thrombin, while citrullination was absent in nucleosomes isolated from non-stimulated eosinophils (**Figure 3A**). We then assessed the effect of AFM-30a and JBI-589, PAD2 and PAD4 inhibitors respectively. EET release in response to both A23187 and PMA was inhibited by JBI-589 with an IC_50_ of 2.7 µM (**Figure 3B**). In these experiments, it was not possible to reach a plateau of maximal inhibitory effect of JBI-589 due to cytotoxicity at concentrations greater than 4 µM. The importance of PAD4-mediated citrullination was further confirmed with two structurally unrelated PAD4 inhibitors GSK484 and BMS-P5 (**Supplementary Figures 4A, B**). The role of PAD2 in EETosis is less pronounced. PMA-induced EETosis was weakly inhibited by AFM-30a (50% reduction at 81.5 µM) and A23187-induced EETosis was not inhibited (**Figure 3C**). Together, these data show that in response to different EETosis stimuli, the EETs released are rich in citrullinated histones and PAD4-mediated citrullination is necessary in the ordered events preceding cell rupture and EET release.

### 3.4 Anti-citrullinated histone antibody CIT-013 potently inhibits EETosis induced by different stimuli

**Figure 4:**
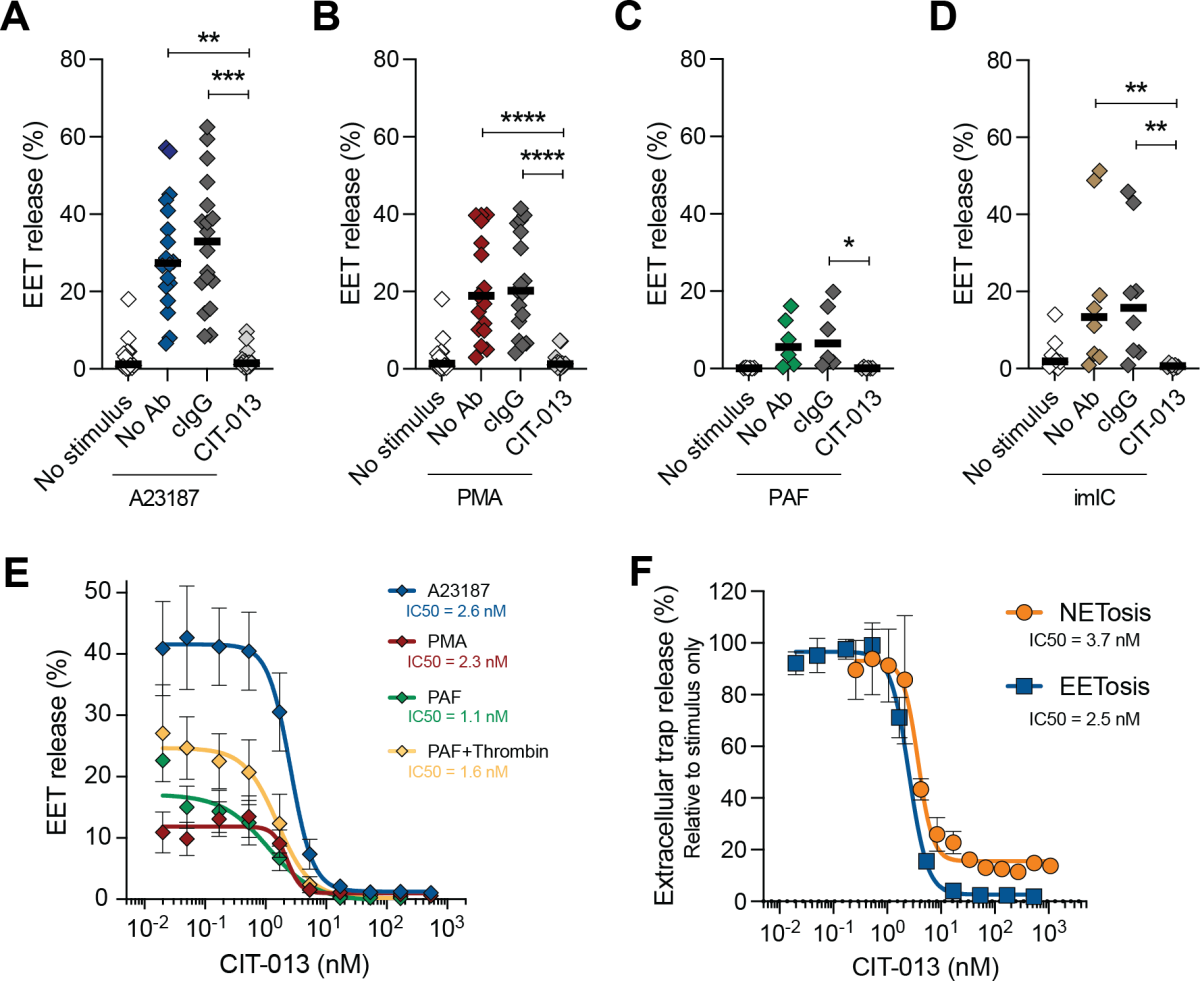
CIT-013 inhibits EET release regardless of the stimulus. Quantification of EET release in the absence (No Ab) or presence of 169.3 nM of either isotype control antibody (cIgG) or CIT-013 at t = 3 h post stimulation with 2 µM A23187 (*n* = 18) **(A)**, 40.5 nM PMA (*n* = 17) **(B)**, 0.25 µM platelet activating factor (PAF) (*n* = 6) **(C)**, and immobilized immune complexes (imICs) (*n* = 8) **(D)**. **(E)** Quantification of EET release induced by A23187, PMA, and 0.5 µM PAF without or with 1 U/mL thrombin at t = 3 h in the presence of different concentrations CIT-013 (*n* = 3-9). **(F)** Quantification of NET and EET release at t = 3 h post stimulation with A23187 in the presence of different concentrations CIT-013 (*n* = 2-9). Data in **(F)** was normalized to stimulus-induced EET or NET release without CIT-013 (set as 100% EET or NET release). *P<0.05, **P<0.01, ***P<0.001 and ****P<0.0001, Friedman test with Dunn’s multiple comparison test **(A, B)**, ordinary one-way ANOVA with Dunnett’s multiple comparison test **(C)**, and Kruskal-Wallis test with Dunn’s multiple comparison test **(D)**.

We have previously described the ability of monoclonal antibody CIT-013 to bind citrullinated histones H2A and H4 with picomolar affinity and block NET release during the final stage of NETosis when plasma membrane integrity was compromised (30). Having shown the presence of citrullinated histones in EETs, we investigated whether CIT-013 could inhibit EET release in a manner similar to that observed in neutrophils. CIT-013 inhibited EET release induced by A23187, PMA, PAF, and imICs (**Figures 4A–D** and **Supplementary Figures 5A–D**) with an IC_50_ in the range of 1-3 nM (**Figure 4E**). The potency of CIT-013 to inhibit EETosis and NETosis in response to A23187 was similar (IC_50_ 2.5 nM and 3.7 nM respectively), as was the percent efficacy in both cell responses (**Figure 4F**). Taken together, these data show that CIT-013 potently blocks EET release with a comparable pharmacology to that shown previously for NET release.

### 3.5 CIT-013 efficiently blocks EET release after loss of plasma membrane integrity

**Figure 5:**
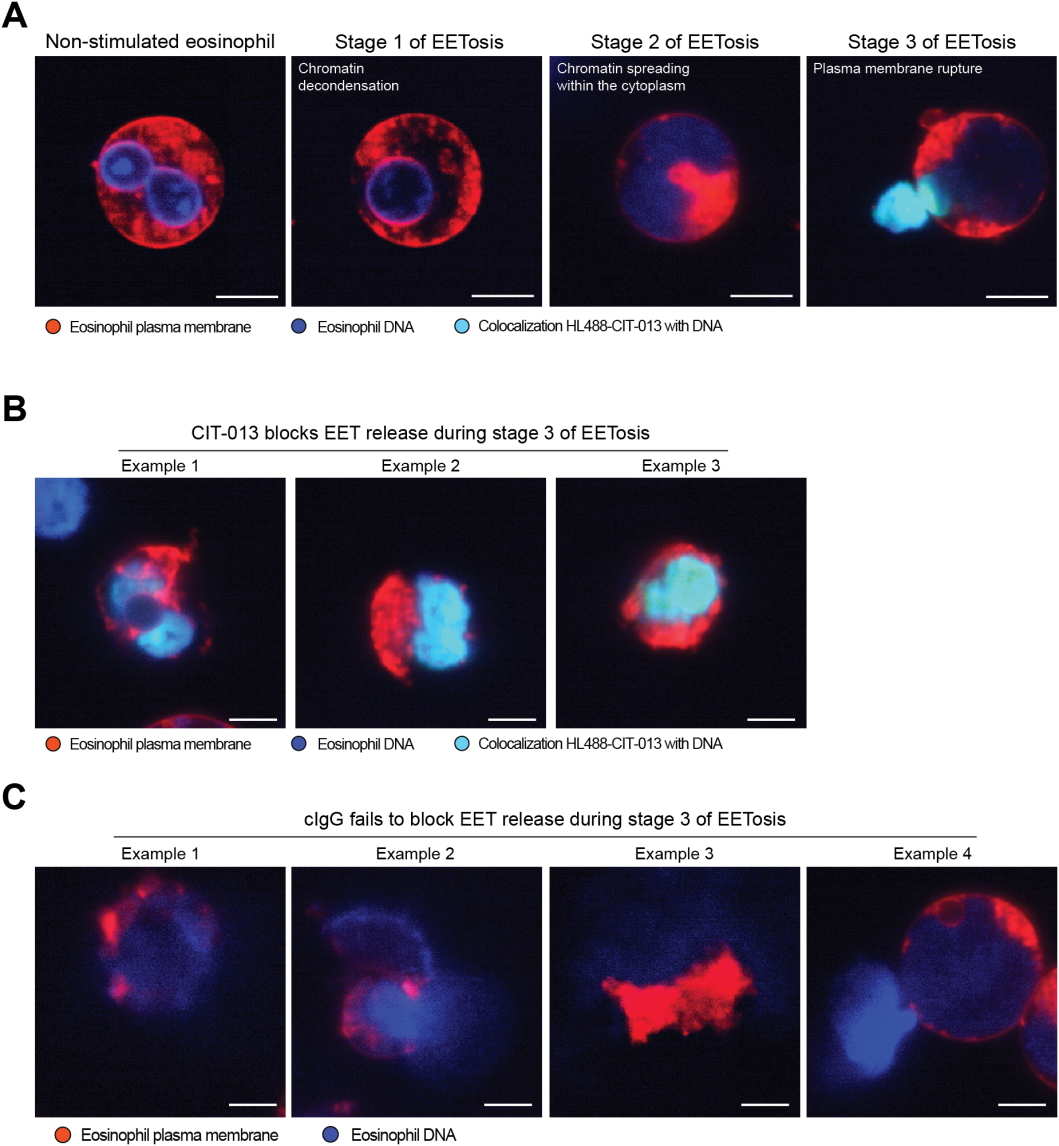
CIT-013 blocks EET release when plasma membrane integrity is compromised. **(A)** Representative immunofluorescence images of a non-stimulated eosinophil or eosinophils triggered for EETosis with 2 µM A23187 in the presence of HiLyte^TM^ Fluor 488-conjugated CIT-013 (HL488-CIT-013). The eosinophils activated for EETosis showed chromatin decondensation and a re-organized rounded nucleus in stage 1 of EETosis, nuclear membrane rupture and chromatin spreading within the cytoplasm in stage 2 of EETosis, and plasma membrane rupture and CIT-013 binding to chromatin in stage 3 of the EETosis pathway. Of note, due to technical limitations we were not able to clearly track one specific eosinophil over time and therefore different eosinophils at each stage of the EETosis pathway are shown. **(B)** More representative immunofluorescence images of eosinophils triggered for EETosis with A23187 in the presence of HiLyte^TM^ Fluor 488-conjugated CIT-013. **(C)** Representative immunofluorescence images of eosinophils triggered for EETosis with A23187 in the presence of HiLyte^TM^ Fluor 488-conjugated isotype control antibody (HL488-cIgG). Scale bars are 10 µm.

Having confirmed that EET release is blocked by CIT-013, we investigated if CIT-013 inhibits EETosis in a manner analogous to its action on neutrophils, i.e., at the final stage of membrane rupture just prior to chromatin expulsion. Eosinophils were stimulated with A23187 and visualized over time with high-magnification live imaging confocal microscopy. CIT-013 binding to stimulated eosinophils was first observed after loss of plasma membrane integrity and exposure of decondensed citrullinated chromatin to the extracellular environment (stage 3 of EETosis; **Figures 5A, B**), while no binding was observed during the earlier stages of EETosis (**Figures 5A** and **Supplementary Video 1**). Binding to EETs and inhibition of EET release was not observed with cIgG (**Figure 5C**). Together, these data demonstrate that histone citrullination offers a point of pharmacological intervention for novel therapeutics targeting inhibition of EETosis.

### 3.6 CIT-013 does not interfere with eosinophil functions other than EETosis

**Figure 6:**
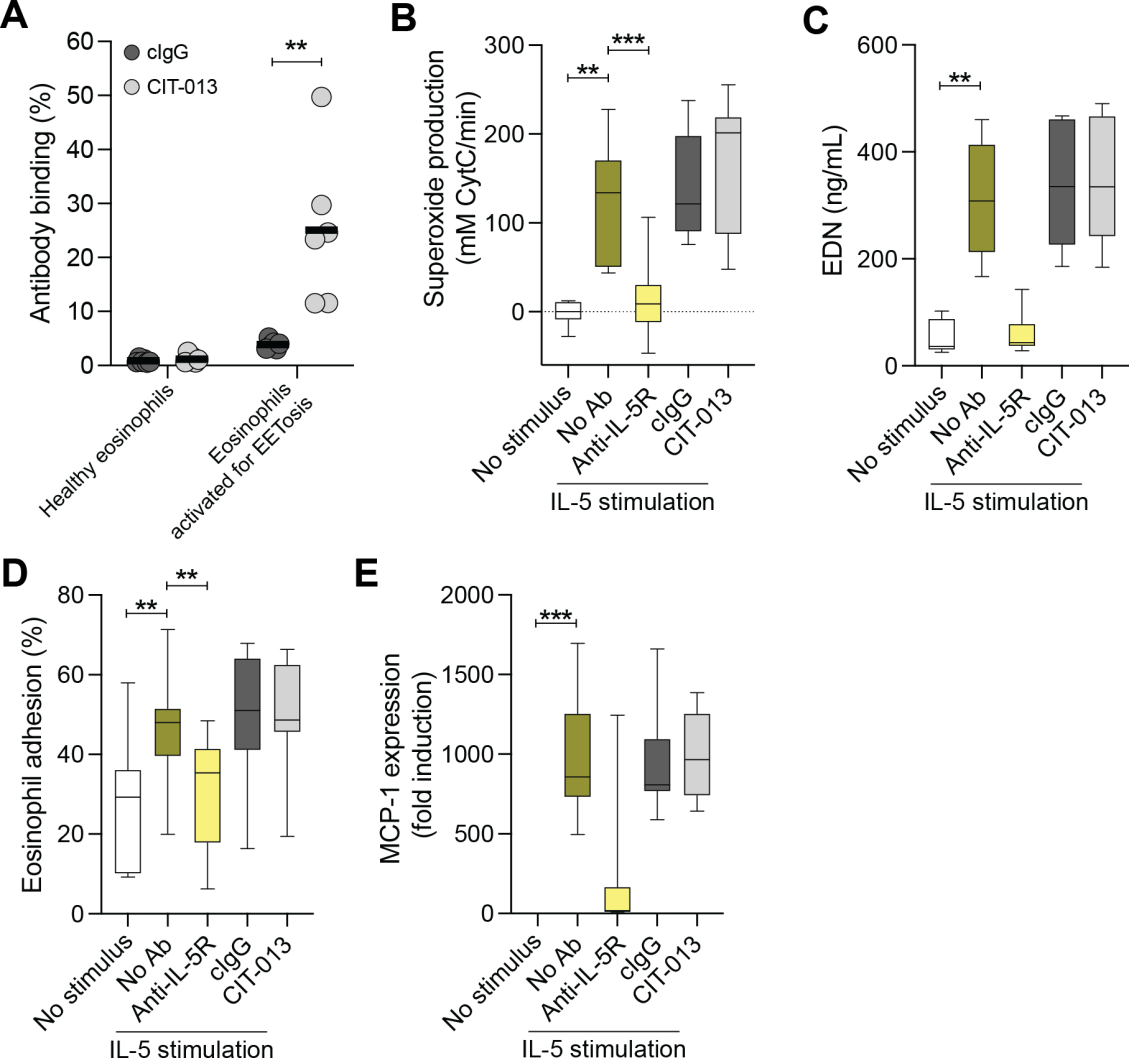
CIT-013 does not inhibit eosinophil functions other than EETosis. **(A)** Binding of HiLyte^TM^ Fluor 488-conjugated isotype control antibody (HL488-cIgG) or HL488-CIT-013 to healthy eosinophils or eosinophils activated for EETosis (*n* = 6). **(B)** Quantification of eosinophil superoxide production induced by 10 ng/mL IL-5 in the absence (No Ab) or presence of 10 µg/mL anti-IL5 receptor antibody (anti-IL-5R), or 169.3 nM of either cIgG or CIT-013 (*n* = 8). **(C)** Quantification of EDN in the supernatant of eosinophil culture medium at t = 4 h post stimulation with IL-5 in the absence or presence of anti-IL-5R, cIgG, or CIT-013 (*n* = 8). **(D)** Quantification of eosinophil adhesion at t = 4 h post stimulation with IL-5 in the absence or presence of anti-IL-5R, cIgG, or CIT-013 (*n* = 7). **(E)** Quantification of MCP-1 gene expression at t = 4 h post stimulation with IL-5 in the absence or presence of anti-IL-5R, cIgG, or CIT-013 (*n* = 8). **P<0.01 and ***P<0.001, Unpaired two-tailed t test **(A)**, RM one-way ANOVA with Tukey’s multiple comparison test **(B, D)**, Kruskal-Wallis test with Dunn’s multiple comparison test **(C, E)**.

We have previously demonstrated that CIT-013 does not bind leukocytes, thrombocytes, and erythrocytes purified from the blood of healthy donors (30). Here we demonstrated that CIT-013 also does not bind healthy eosinophils in the absence of an EETosis stimulus (**Figure 6A**). We further investigated the possibility of CIT-013 inhibiting eosinophil responses other than EETosis. While the positive control anti-IL-5 receptor blocking antibody benralizumab reduced all studied eosinophil responses to IL-5, CIT-013 did not interfere with IL-5-induced superoxide production (**Figure 6B**), degranulation (**Figure 6C**), eosinophil adhesion (**Figure 6D**) or MCP-1 cytokine expression (**Figure 6E**). Eosinophil degranulation in response to A23187, PMA, and PAF was also not affected by CIT-013 (**Supplementary Figure 6**). These data indicate that targeting EET release with an anti-citrullinated histone antibody enables a highly specific inhibition of EETosis, with all other innate functions remaining intact.

### 3.7 Galectin-10 release correlates with EET release but is independent of citrullination

**Figure 7:**
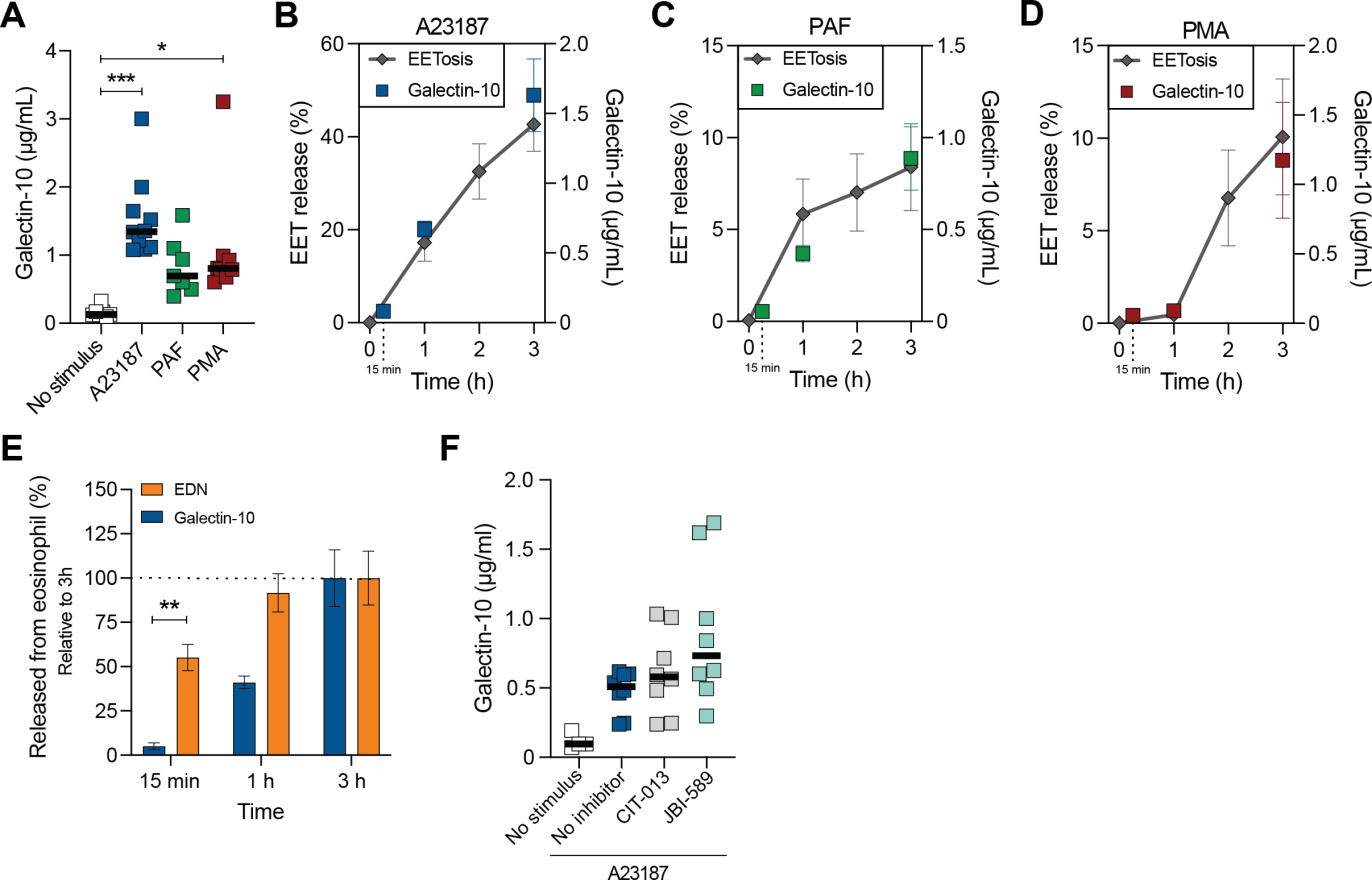
Galectin-10 release is independent of citrullination and chromatin release during EETosis. **(A)** Quantification of galectin-10 release in the supernatant of eosinophil culture medium at t = 3 h post stimulation of EETosis-inducing stimuli 2 µM A23187, 40.5 nM PMA, and 0.5 µM platelet activating factor PAF (n = 5-8). Combined kinetic profile of EET release and galectin-10 release at t = 0, 1, 2, and 3 h (EETs) or t = 15 min, 1 h, and 3 h (galectin-10) post stimulation with A23187 (n = 7-12) **(B)**, PAF (*n* = 5-12) **(C)**, and PMA (n = 5-11) **(D)**. **(E)** Galectin-10 and EDN release in the supernatant of eosinophil culture medium at t = 15 min, 1 h, and 3 h post stimulation of A23187 (relative to the release at t = 3 h (=100%); n = 7-8). **(F)** Quantification of galectin-10 release in the supernatant of eosinophil culture medium at t = 3 h post stimulation with EETosis-inducing stimuli A23187 in the absence (No inhibitor) or presence of EETosis inhibitors 169.3 nM CIT-013 and 4 µM JBI-589. *P<0.05, **P<0.01, and ***P<0.01, Kruskal-Wallis test with Dunn’s multiple comparison test **(A)** and two-way ANOVA with Sidak’s multiple comparisons test **(E)**.

Eosinophils highly express pro-inflammatory galectin-10 juxtaposed to the plasma membrane and its release does not occur through secretory pathways, but rather during plasma membrane rupture (41, 42). EETosis-inducing stimuli have been previously described to induce galectin-10 release (26, 42, 43), however it is unknown whether pathways associated with release of EETs and galectin-10 overlap. To address this, we first investigated the kinetics of galectin-10 release following different EETosis stimuli. Elevated levels of extracellular galectin-10 were detected 3 hours after stimulation with A23187, PAF, or PMA (**Figure 7A**) with overlapping kinetics of galectin-10 and EET release for each stimulus (**Figures 7B–D**). Degranulation, as quantified with EDN release, occurred prior to galectin-10 release (<15min) (**Figure 7E**).

To assess whether inhibitors of EETosis also block the release of galectin-10, eosinophils were activated with various EETosis stimuli in the presence or absence of CIT-013 or PAD4 inhibitor JBI-589. Galectin-10 was measured in the supernatant after 3 hours. Although CIT-013 and JBI-589 efficiently blocked expulsion of EETs into the extracellular environment upon stimulation with A23187, PMA, and PAF (**Figures 3B**, **4A–C**), galectin-10 release was not inhibited (**Figure 7F**; **Supplementary Figure 7**). These data demonstrate that galectin-10 is released with a similar kinetic profile as EETosis upon stimulation with various stimuli but it occurs in a citrullination-independent manner.

## 4 Discussion

Accumulating evidence suggests that EETs may act as pathogenic factors for eosinophil-driven disease, however there are currently no direct EETosis targeting therapies. This study provides new insights into the requirement of citrullination for EETosis and the pharmacology of EETosis inhibition with a monoclonal antibody targeting citrullinated histones H2A and H4. This study contributes to a better understanding of the kinetics and magnitude of EET release from healthy volunteer eosinophils in response to several stimuli and verify that PAD4-mediated citrullination is required for EETosis. The kinetics of degranulation is shown to be more rapid than EETosis and while the timing of galectin-10 release overlaps with EETosis, the galectin-10 signaling pathway is different from EETosis with its release being independent of citrullination.

The feasibility of targeting histone citrullination for EETosis inhibition is emphasized by the observation that small molecule inhibitors of PAD4 and CIT-013 potently and highly efficacious inhibit EET release. The specificity of CIT-013 inhibition of EETosis is demonstrated through its lack of influence on other eosinophilic functions such as degranulation, adhesion, superoxide production and cytokine expression. Finally, the detection of abundant citrullinated histones colocalizing with EETs in tissue of ECRS patients underscores the therapeutic potential of CIT-013, PAD inhibitors and other citrullination targeting therapeutics for eosinophilic diseases with unmet need.

The presence of EETs has been demonstrated in thrombosis (11, 44) and several eosinophilic inflammatory disorders such as eosinophilic granulomatosis with polyangiitis (EGPA) (42, 45), eosinophilic bronchiolitis (46), allergic bronchopulmonary aspergillosis (ABPA) (14, 26, 47), sialodochitis fibrinosa (48) and hyper eosinophil syndrome (HES) (49). Furthermore, EETs have been described in tissue biopsies or allergic mucin of ECRS patients (8, 9, 18, 21–23, 25) and these findings correlate with clinical symptoms (18, 22) and may have prognostic utility (24). Besides the abundant evidence of the presence of EETs in inflamed tissue, it becomes clear that EETs contribute to inflamed pathology. In a preclinical mouse model, inhaled administration of EETs augmented induced pulmonary inflammation in lung tissue which was characterized by epithelial activation and an exacerbated type 2 immune response (50, 51). A preclinical model of atrial thrombosis also demonstrated that eosinophils induce the development of thrombi by releasing EETs with pharmacological inhibition of EETosis decreasing thrombus stability (44). This indicates that EETs contain pro-inflammatory characteristics contributing to the pathophysiology of eosinophil-driven diseases and may offer a novel target for anti-inflammatory approaches.

Eosinophilic inflammatory disorders are often treated with corticosteroids, but long-term treatment is limited due to poor tolerability (52). Biological agents developed to treat type 2 eosinophilic disorders target IL-5, IL-13, IL-33, IL-5 receptor (IL-5R), IL-4Ra, IgE, thymic stromal lymphopoietin (TSLP), or sialic acid-binding Ig-like lectin 8 (Siglec-8) and thereby affect either eosinophilic viability or suppress multiple eosinophil functions (5, 53–55). There remains a need for differentiated approaches targeting specific aspects of eosinophil-mediated inflammation, like EETosis. Based on the presence of CIT-013’s epitope in tissue from patients with an eosinophilic inflammatory disease and similar EETosis-inhibiting mechanism to that of NETosis (30), it can be envisioned, as described previously for NETs, that CIT-013 will be capable of inhibiting EET release and enhancing clearance of tissue EETs by macrophages. The specific effect on EETosis, while leaving other innate eosinophil functions intact is an advantage of CIT-013 that may reduce risk of increased infection rates associated with certain therapies (56). Furthermore, it is reasonable to hypothesize that CIT-013’s capability to target citrullinated histones will be agnostic to the cellular origin of the ETs and may present a beneficial therapy in disorders that have mixed eosinophil/neutrophil pathologies, such as severe asthma (57), CRS (24, 58), atherosclerosis and thrombosis (44, 59).

Eosinophilic inflammatory disorders are characterized by the presence of CLCs at sites of inflammation formed as a result of galectin-10 crystallization (26). Although galectin-10 has been described as an immunoregulatory molecule by suppressing T-cell proliferation (60), CLCs are pro-inflammatory in nature and induce NLRP3 inflammasome activation, promotion of proinflammatory cytokine production, neutrophil and monocyte influx, and boost the humoral immunity (28, 61). The presence of CLCs has been associated with the accumulation of tissue EETs in eosinophilic diseases (22, 23, 26) and eosinophil extracellular traps augment CLC formation (22). Despite CIT-013 and PAD4 inhibitors not inhibiting galectin-10 release, blocking EETosis may suppress the crystallization of extracellular galectin-10 into pro-inflammatory CLCs and thereby preserve the immunoregulatory properties of galectin-10. Further studies focusing on CLCs are needed to clarify whether CLC formation is indeed affected by CIT-013’s action.

A limitation of the current study is that the characteristics of EETosis inhibition by CIT-013 and PAD4 inhibitors were only tested *in vitro*. Though we have shown beneficial effect with a mouse version of CIT-013 in numerous animal models of neutrophilic inflammation, it would be valuable to test the effect of EETosis inhibition by targeting citrullination in a preclinical model that predominantly features eosinophilic, EETosis mediated inflammation.

In conclusion, our findings emphasize that citrullination is required for EETosis and that citrullinated EETs are abundantly present in tissue characterized by eosinophilic inflammation. This research provides new insights into the pharmacology of EETosis inhibition and identifies the potential of targeting citrullination as a therapeutic strategy for eosinophil-driven diseases with unmet need.

## Data Availability

The original contributions presented in the study are included in the article/**Supplementary Material**, further inquiries can be directed to the corresponding author/s. All data are available under a material transfer agreement with Citryll B.V.
