# Supplementary material for: Inhibition of EETosis with an anti-citrullinated histone antibody: a novel therapeutic approach for eosinophilic inflammatory disorders
Source: Front Immunol. 2025 Feb 20;16:1533407. doi: 10.3389/fimmu.2025.1533407 (PMC11882434; doi:10.3389/fimmu.2025.1533407)
Supplement: Supplementary file 1 [file DataSheet1.pdf]

Supplementary material

Supplementary video

Representative movie of CIT-013-mediated inhibition of EET release in an eosinophil that was stimulated with A23187 in the presence of HiLyte^{TM} Fluor 488-conjugated CIT-013. Eosinophil plasma membrane (red), eosinophil DNA (blue), and CIT-013 (green). Magenta arrow indicates the site of CIT-013 binding to DNA (cyan).

Supplementary figures

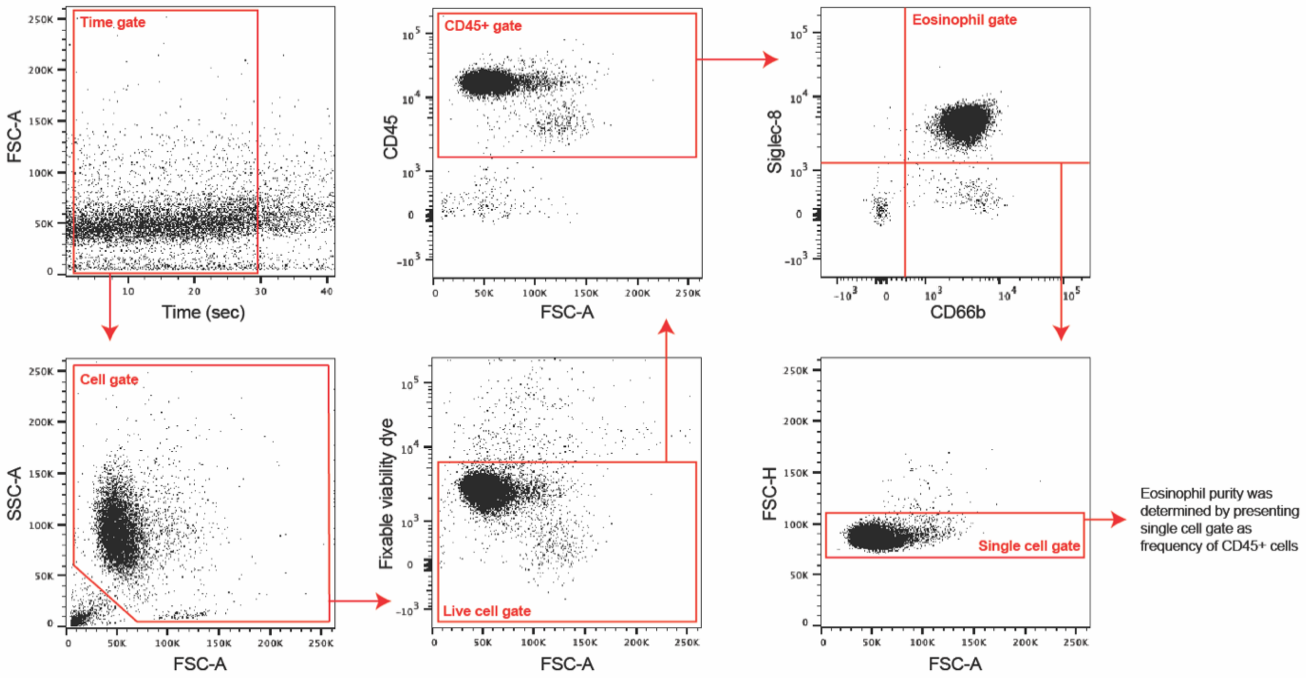

Supplemental figure 1 | Eosinophil purity check after isolation procedure from venous blood of healthy individuals. The following gating strategy was used: 1. time gate, 2. cell gate in forward- and side-scatter, 3. live cell gate with fixable viability dye, 4. CD45+ cell gate, 5. Siglec-8+ and CD66b+ cell gate (eosinophils), and 6. single cell gate in forward-area and forward-height scatter. Eosinophil purity was determined by plotting the single cells as frequency of CD45+ cells.

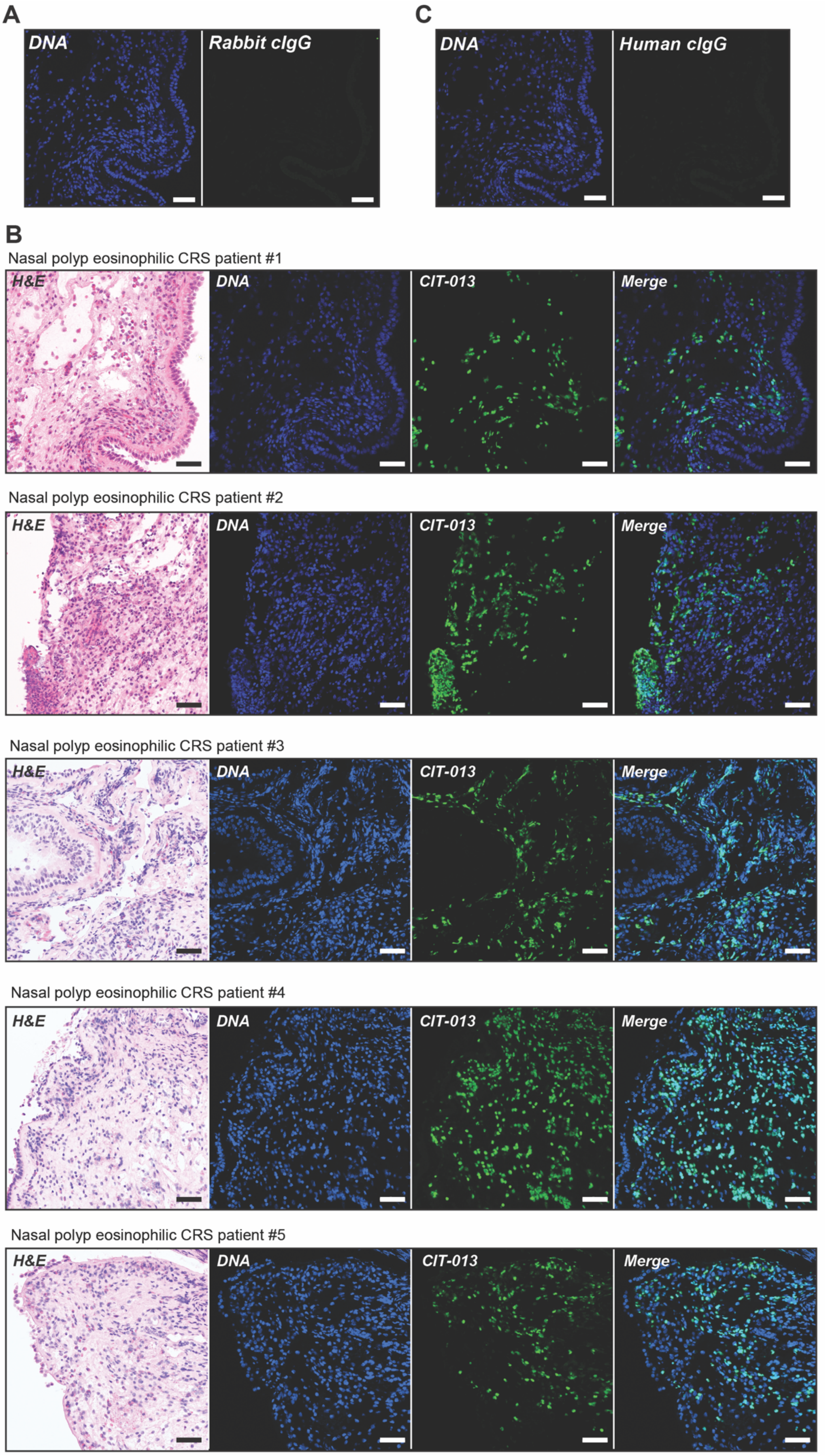

Supplemental figure 2 | (A) Immunofluorescence images of nasal polyp tissue from ECRS patients showing DNA (blue) and rabbit isotype control antibody (Rabbit cIgG). **(B)** Representative images of nasal polyp tissue from five ECRS patients stained with hematoxylin and eosin (H&E), DNA (blue), and FITC-conjugated CIT-013 (green). **(C)** Immunofluorescence images of nasal polyps tissue from ECRS patients showing DNA (blue) and FITC-conjugated human isotype control antibody (Human cIgG). Scale bars are 50 µm.

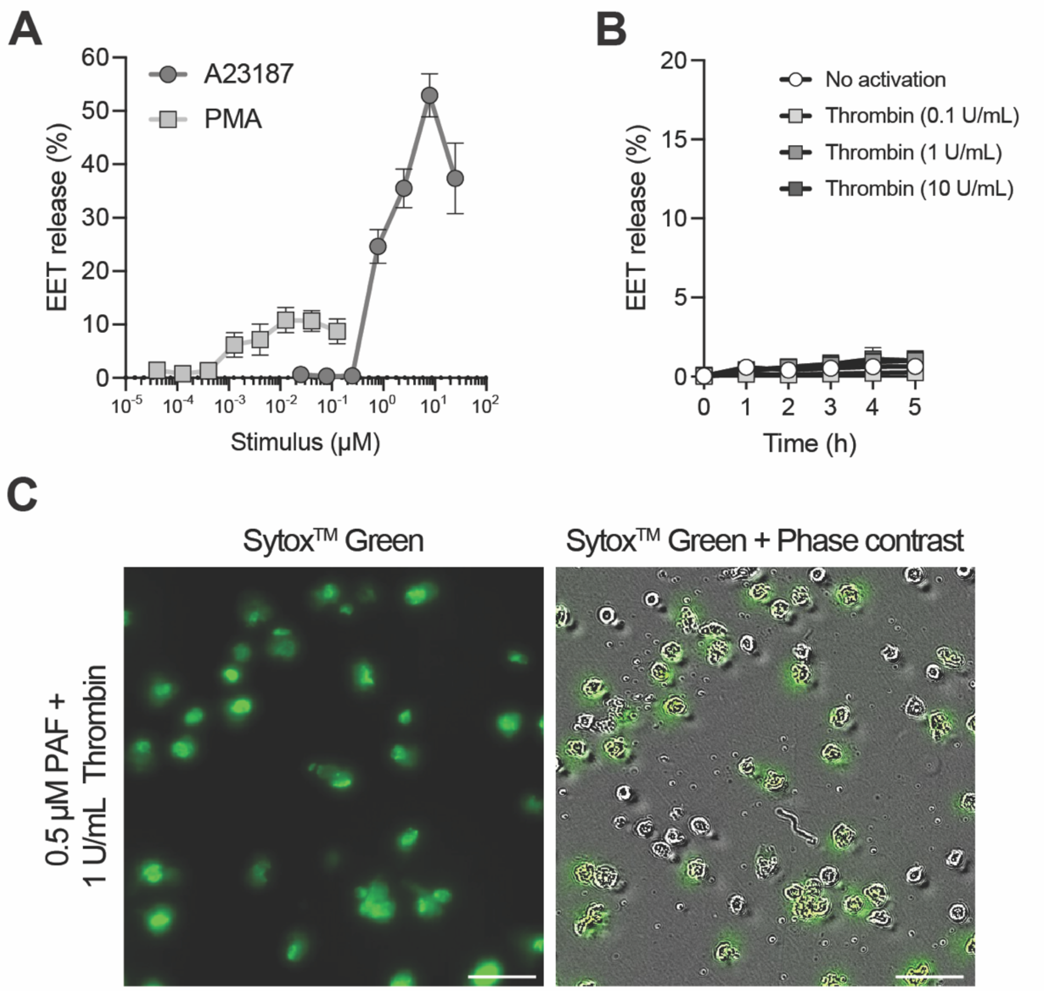

Supplemental figure 3 | (A) Quantification of EET release at t = 3 h upon stimulation with different concentrations of PMA and A23187 (*n* = 3-8). **(B)** Quantification of EET release over time upon stimulation with indicated concentrations of thrombin (*n* = 5-8). **(C)** Representative images of EET release at 3 h post stimulation with 0.5 μM PAF in combination with 1 U/mL thrombin. Scale bars are 50 μm.

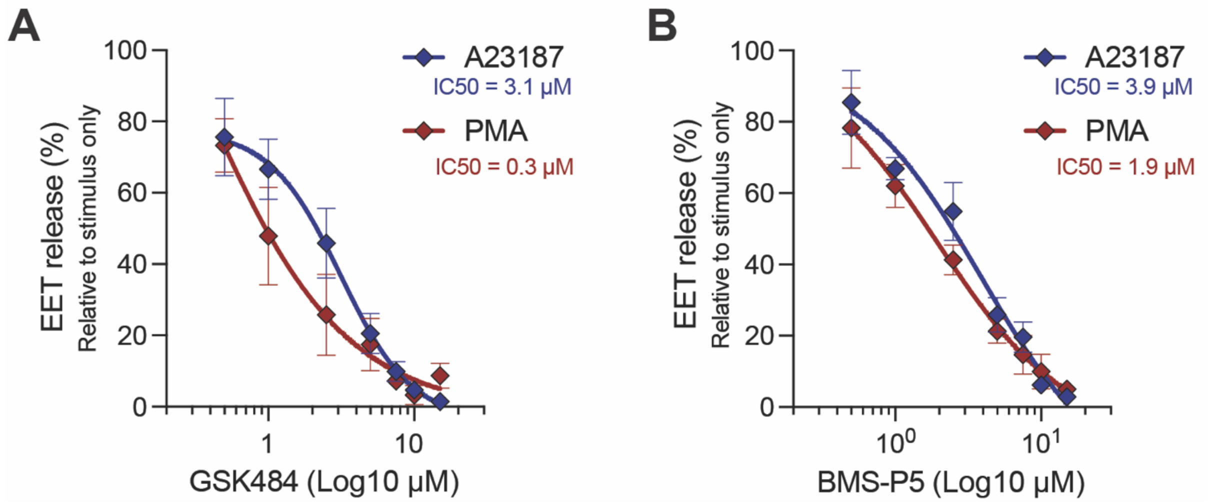

Supplemental figure 4 | Quantification of 2 μM A23187- and 40.5 nM PMA-induced EET release at t = 3 h in the presence of different concentrations of GSK484 **(A)** or BMS-P5 **(B)**, both inhibitors of PAD4 (*n* = 4). Data were normalized to A23187- or PMA-induced EET release without chemical inhibitor (set as 100% EET release).

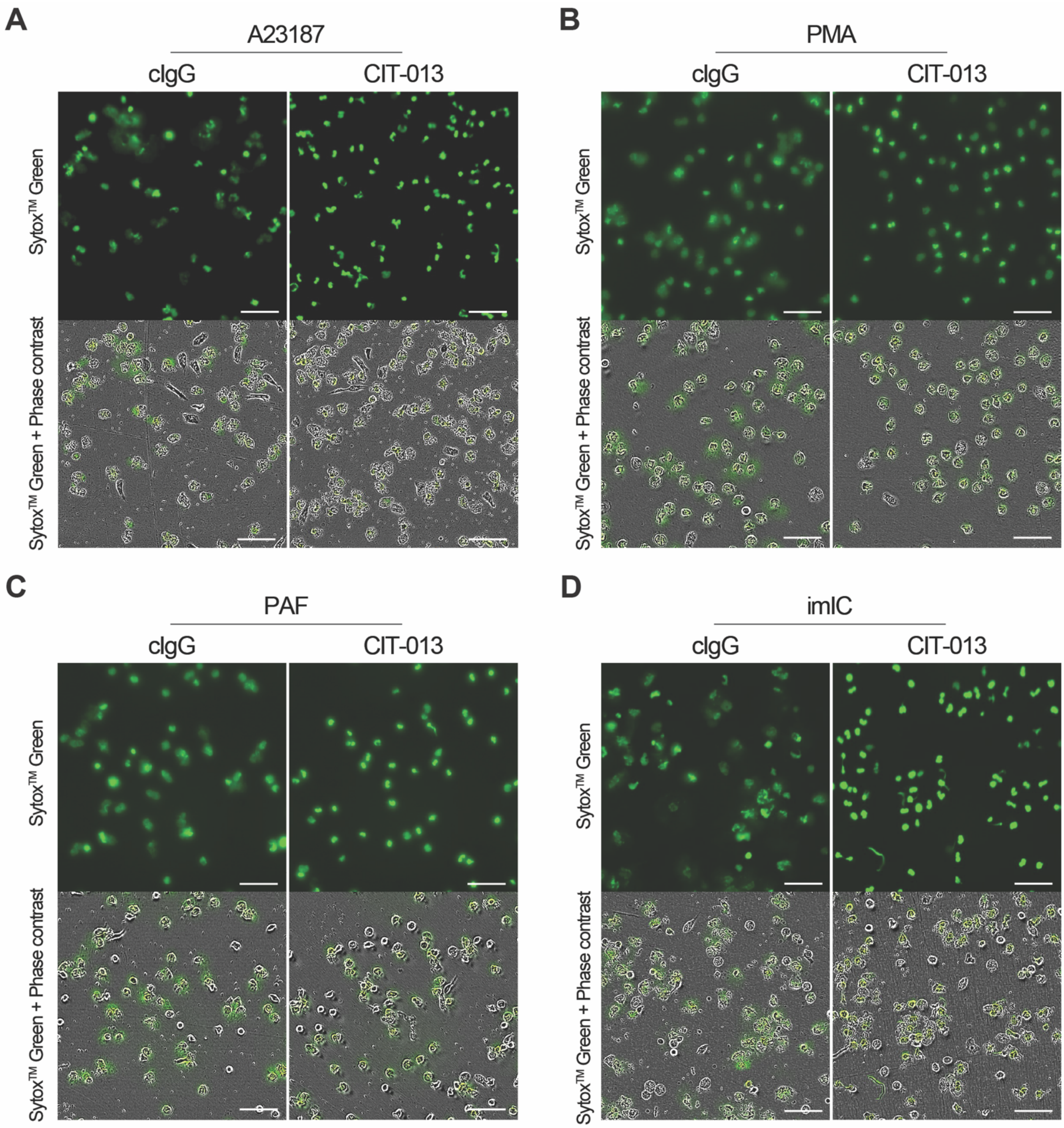

Supplemental figure 5 | Representative images of EET release inhibition at t = 3 h with 2 µM A23187 **(A)**, 40.5 nM PMA **(B)**, 0.25 µM platelet activating factor (PAF) **(C)**, and immobilized immune complexes (imIC) **(D)** in the presence of 169.3 nM of either isotype control antibody (cIgG) or CIT-013. Scale bars are 50 µm.

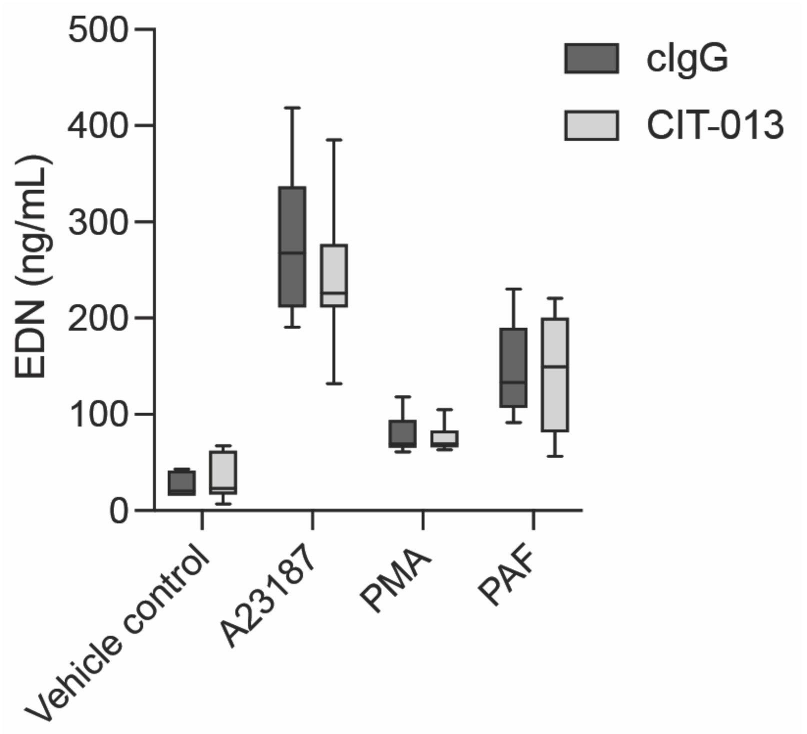

Supplemental figure 6 | Quantification of EDN in the supernatant of eosinophil culture medium at t = 3 h post stimulation with 2 µM A23187, 40.5 nM PMA, and 0.25 µM PAF in the presence of 169.3 nM of either isotype control antibody (cIgG) or CIT-013.

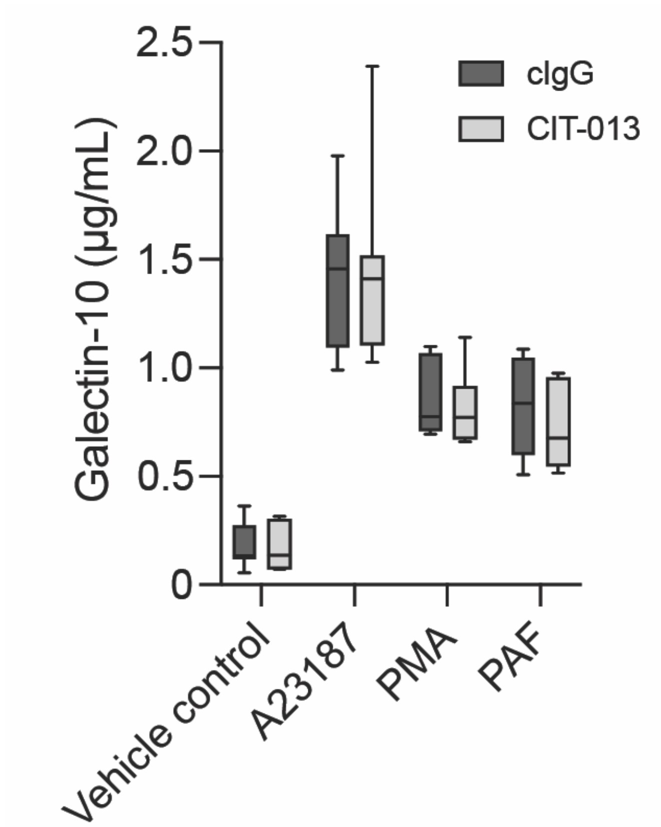

Supplemental figure 7 | Quantification of galectin-10 in the supernatant of eosinophil culture medium at t = 3 h post stimulation with 2 µM A23187, 40.5 nM PMA, and 0.5 µM PAF in the presence of 169.3 nM of either isotype control antibody (cIgG) or CIT-013.
